# Genomic location of stripe rust resistance in a hexaploid derivative of durum wheat Glossy Huguenot and development of closely linked markers

**DOI:** 10.1038/s41598-025-17328-z

**Published:** 2025-09-26

**Authors:** Hongyu Li, Geetha Perera, Debbie Wong, Kerrie Forrest, Xiaodi Xia, Chunhong Chen, Matthew Hayden, Urmil Bansal, Harbans Bariana, Rohit Mago

**Affiliations:** 1 CSIRO Agriculture and Food, Clunies Ross Street, Box 1700, Canberra, ACT 2601 Australia; 2 Agriculture Victoria, Agriculture Research Division, AgriBio, Centre for AgriBioscience, Bundoora, VIC 3083 Australia; 3 Plant Breeding Institute, School of Life and Environmental Sciences, Faculty of Science, The University of Sydney, 107 Cobbitty Road, Cobbitty, NSW 2570 Australia; 4 School of Plant Sciences, Hawkesbury Campus, Western Sydney University, 10 Bourke Street, Richmond, NSW 2753 Australia

**Keywords:** Genetics, Plant sciences

## Abstract

Stripe rust, caused by *Puccinia striiformis* f. sp. *tritici* (*Pst*), is a devastating fungal disease that affects wheat production in many regions of the world. The identification and characterisation of new sources of host plant resistance is required to enrich the existing gene pool. Durum wheat landrace Glossy Huguenot showed high level of resistance to stripe rust in the field. To utilise this resistance in wider wheat germplasm, we transferred it to common wheat cultivar Westonia. A backcross_2_F_5_ (BC_2_F_5_) line (WGH54) which showed high levels of all stage resistance against the then prevalent *Pst* pathotypes was crossed with the susceptible parent Avocet S (AvS) and F_2:3_ generation was raised. Monogenic segregation was observed among WGH54/AvS F_2:3_ families. Bulked segregant analysis using iSelect wheat 90 K Infinium SNP array mapped the stripe rust resistance on chromosome 2A. The gene was temporarily named as *YRWGH54*. Single nucleotide polymorphism (SNP) markers were used to refine the location of *YRWGH54*. Genotyping showed chromosomal rearrangements in this genomic region when compared with the Chinese Spring (CS) reference sequence. Stripe rust resistance gene *YR32* was located on chromosome 2AL previously and markers linked with it were mapped in the same region as *YRWGH54*. Greenhouse tests with recent *Pst* pathotypes showed same virulence/avirulence specificity suggesting that *YRWGH54* and *YR32* may be the same. Closely linked KASP markers identified in this study will be useful for marker assisted pyramiding of *YRWGH54* with other marker-tagged stripe rust resistance genes in future wheat cultivars to achieve durable control.

## Introduction

Stripe rust, caused by *Puccinia striiformis* f. sp. *tritici* (*Pst*), causes yield losses in wheat crops worldwide^1^. In 2007, up to 90 million dollars were spent on fungicide applications to control stripe rust in Australia^2^. Chemical control of rust diseases leads to environmental pollution and adds a significant extra cost to wheat production^3^. The release of resistant wheat cultivars provides an economical and “environmentally friendly” means to control stripe rust.

Stripe rust resistance in wheat falls under two categories: All stage resistance (ASR) and adult plant resistance (APR). ASR genes provide high levels of resistance across all plant growth stages; however, evolution in pathogen populations often renders such genes ineffective short time after their deployment. In contrast, APR genes express at post-seedling stages, impart partial resistance^3,4^ and are generally non-race specific. A single APR gene does not condition commercially acceptable level of resistance. Hence pyramiding of race specific ASR genes with non-race-specific APR genes into individual cultivars makes resistance to last longer^5,6^. Therefore, identification and characterization of new sources of ASR and APR is important to achieve genetic diversity for resistance among new wheat cultivars.

Although more than 80 stripe rust resistance (*YR*) loci have been genetically mapped to wheat chromosomes, only a small number of these genes have been isolated so far (*YR10*, *YR18*, *YR36*, *YR46*, *YR5*/*YRSP*, *YR7, YR15* and *YR27*). Among these, six genes belong to the ASR class, five genes (*YR10*, *YR5*/*YRSP*, *YR7*and *YR27*) encode nucleotide-binding site leucine-rich repeat proteins (NLR)^7–9^ and one gene (*YR15*) encodes a putative kinase-pseudokinase protein^10^. Two pleiotropic APR genes *YR18*/*LR34*/*SR57* (*YR18*) and *YR46*/*LR67*/*SR55* (*YR46*) provide multi-pathogen resistance. The gene *YR18* encodes an ATP-binding cassette (ABC) transporter protein^11,12^, while *YR46* encodes a defective hexose transporter^13^. *YR36* only gives resistance to stripe rust and encodes a kinase steroidogenic acute regulatory protein-related lipid transfer (START) domain protein^14^.

The evolution among *Pst* to evolve rapidly with increased virulence spectrum has overcome many major resistance genes and even some APR genes present in wheat varieties^15^. Availability of genetically diverse and effective resistance genes is the prerequisite for their pyramiding in new wheat cultivars. The durum wheat landrace Glossy Huguenot (GH) showed resistance to then prevalent *Pst* pathotypes both in glasshouse and field conditions. To use this resistance in wider bread wheat germplasm, GH was crossed to a susceptible wheat variety Westonia and a BC_2_F_5_ line, WGH54, was found to be highly resistant at the seedling and adult plant stages. This study describes the chromosomal location of all stage stripe rust resistance carried by WGH54 and development of high throughput markers linked with resistance for its marker assisted selection in wheat breeding programs.

## Materials and methods

### Plant material and development of a mapping population

Durum wheat landrace Glossy Huguenot was crossed with stripe rust susceptible bread wheat cultivar Westonia and the F_1_ was backcrossed to recurrent parent Westonia to develop backcross _2_F_5_ (BC_2_F_5_) lines. A BC_2_F_5_ line WGH54 which showed high level of resistance to *Pst* pathotype 134 E16A + 17 + , prevalent at the time was selected for further investigation. The mapping population used in this study was generated by crossing WGH54 with the susceptible wheat Avocet S (AvS). The initial population consisted of 136 F_2_ plants and an additional 88 F_2_ plants were grown later. Twenty-four F_2:3_ recombinants were identified by screening with flanking markers KASP-IWB7664 and KASP-IWB13648. Seeds of wheat cultivar Carstens V which carries *YR32* was obtained from Australian Grain Genebank (AGG).

A set of 73 wheat cultivars were used to validate markers linked with the ASR gene carried by WGH54.

### Stripe rust screening in greenhouse and field

The WGH54/AvS F_2_ (total 224) and 24 F_2:3_ families were tested for stripe rust response at the two-leaf stage. Seedlings were raised in growth chambers at 23 °C/18 °C for 16 h/8 h day/night until two leaf stage. A mixture of urediniospores of *Pst* pathotype 134 E16A + Yr17 + (one part) and talcum powder (3parts) was applied gently on leaves with the help of a paint brush^16^. Infected plants were kept in a plastic box to maintain high humidity and incubated in a climate chamber at 10 °C and 90% humidity for 24 h in dark. Plants were then transferred to a climate chamber at 18 °C/18 °C for 16 h/8 h day/night. The infection type (IT) variation was scored after 14 days of inoculation on a 0–4 scale described in McIntosh et al.,^17^. ITs 0–2 were classified as resistant and ITs 3–4 were classified as susceptible. WGH54 was tested in field trials in 2017 at the Plant Breeding Institute, University of Sydney, NSW, Australia. Plants were inoculated with a mixture of *Pst* pathotypes 134 E16A + Yr17 + Yr27 + , 134 E16A + YrJ + YrT + , 150 E16A + and 110 E143A + (2017). Adult plant stripe rust response variation under field conditions was recorded using the 1–9 scale described by Bariana et al.^18^.

*Pst* pathotypes 239 E237A-Yr17 + Yr33 + and 198 E16A + YrJ + YrT + Yr17 + which appeared in 2019 and 2021, respectively, were used for phenotyping the parents in the greenhouse.

## Molecular analyses

### DNA extraction

DNA was extracted from 10-day-old leaves of glasshouse grown material using method described in Yu et al.^19^. We checked DNA quality and quantity on a 0.8% agarose gel and with a NanoDrop spectrophotometer (Thermo Scientific).

### Genotyping with linked SNP and simple sequence repeat (SSR) markers

DNA from10 resistant and 10 susceptible seedlings of WGH54/AvS F_2_ were genotyped using the Illumina iSelect 90 K Infinium SNP genotyping array^20^ to determine chromosomal location of the resistance locus. Resistance-linked SNP loci were then converted to Kompetitive Allele Specific PCR (KASP) markers and were assayed on the entire F_2:3_ population. KASP markers were amplified using the CFX96 Real Time System (Biorad, USA) and KASP master mix (LGC, UK). Primers used for KASP marker analysis were obtained from Polymarker (http://www.polymarker.info/designed_primers). Primer sequences for SSR markers were obtained from Graingenes database (https://wheat.pw.usda.gov/GG3/). Primer sequences of KASP markers are provided in supplementary Table S1.

### Data analysis

Goodness of fit of observed segregation data to the expected genetic ratios was tested through Chi-squared analysis. BLAST searches against the Chinese Spring genome sequence were undertaken on the wheat 10 genomes website (https://galaxy-web.ipk-gatersleben.de/). Chinese Spring gene models were obtained from https://urgi.versailles.inra.fr/blast/?dbgroup=wheat_all&program=blastn portal using J Browse^21^.

## Results

**Fig. 1:**
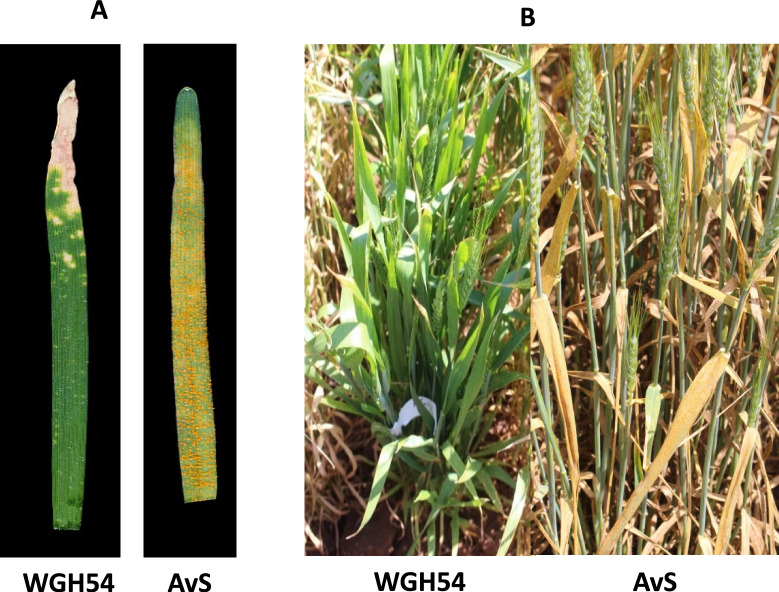
Stripe rust responses of WGH54 and Avocet S (AvS): (**A**) seedling; (**B**)**.** adult plants in the field.

**Fig. 2:**
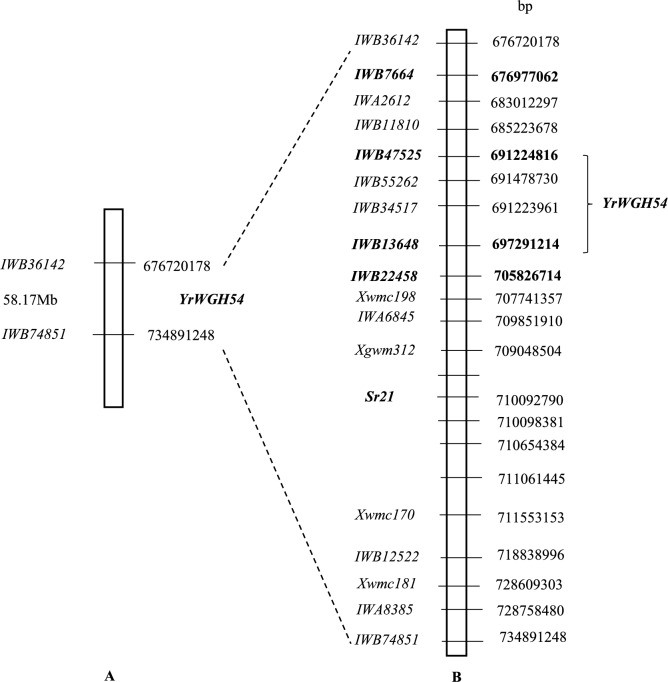
Chromosome 2AL linkage map of WGH54/Avocet S showing location of *YRWGH54:* (**A**) Initial SNP map of WGH54/AvS based on 90 K SNP array bulked segregant analysis. (**B**) high-density map of WGH54/AvS. Marker order is based on position in CSv1.0 reference sequence.

Bread wheat line WGH54 exhibited infection type (IT) ;N at the seedling stage, when tested against *Pst* pathotype 134 E16A + Yr17 + Yr27 + and the susceptible parent AvS showed IT 3 + (Fig. 1 A, Table 1). Adult plant response of WGH54 was consistently scored lower (score 4) than the susceptible parent Avocet S (8–9) in the field in 2017 (Fig. 1B). Phenotyping of WGH54/AvS F_2_ population showed monogenic segregation for stripe rust resistance (97 resistant plants and 39 susceptible plants, (χ^2^_3:1_ = 0.98, *P* = 0.3221). The underlying resistance locus was temporarily named *YrWGH54*.The 90K iSelect Illumina SNP array was used to genotype 10 resistant and 10 susceptible WGH54/AvS-derived F_2_ plants and both parents. Seventy SNP markers from the long arm of chromosome 2A showed linkage with the stripe rust resistance gene *YRWGH54*. These markers are located within the 58.17Mbp region (nucleotides 676,720,178–734,891,248 of the Chinese Spring v 1.0 reference genome) on chromosome 2AL (Fig. 2A).

**Table 1:** Infection types produced by wheat genotypes against three pathotypes of *Puccinia striiformis* f. sp. *tritici*.

Wheat	134 E16A + Yr17 + Yr27 +	239 E237 A-Yr17 + Yr33 +	198 E16 A + YrJ + YrT + Yr17 +
Avocet S	3 +	3 +	3 +
WGH54	; to ;n	3 +	;C
Carstens V (*Yr32*)	;C	3 +	;C

To refine the chromosome location of *YRWGH54*, 12 KASP markers (*KASP-IWB36142, KASP-IWB7664, KASP-IWB77174, KASP-IWB11810, KASP-IWB47525, KASP-IWB55262, KASP-IWB13648, IWA6845, KASP-IWB12522, KASP- IWA1960, KASP-IWA8385, KASP-IWB74851*) between positions 676,720,178–734,891,248 (on CS v1.0) were polymorphic between parents and were used for genotyping the entire F_2_ population. An additional 88 F_2:3_ plants from a single heterozygous F_2_ plants were also included for genotyping. *YRWGH54* was placed between markers *KASP-IWB47525* and *KASP-IWB13648*. An expected Mendelian segregation of 1:2:1 for markers linked with *YRWGH54* was observed among F_2:3_ plants (Table 2). Based on this analysis *YRWGH54* was placed in a 6.0Mbp region with reference to CS V1.0 genomic sequence (Fig. 2 B).

**Table 2:** Segregation of markers in WGH54/AvS F_2:3_ population.

KASP marker	Lines evaluated	Observed ratio*	Expected ratio	χ2	P-Value
*IWB36142*	88	23A:43H:22B	1:2:1	0.068	0.966
*IWB7664*	88	24A:42H:22B	1:2:1	0.273	0.873
*IWB77174*	88	27A:37H:24B	1:2:1	2.432	0.296
*IWB11810*	88	27A:36H:25B	1:2:1	3.000	0.223
*IWB22730*	88	26A:37H :25B	1:2:1	2.250	0.325

*A = Resistant, H = Heterozygous, B = Susceptible.

To increase the marker density in this region and fine map *YRWGH54*, additional markers *KASP-IWB22730, KASP-IWB46979, KASP-IWB43829, KASP-IWB58832, IWA1275, KASP-IWB1036, KASP-IWB78803, KASP-IWB32429, KASP-IWB40126, KASP-IWB43663* and *KASP-IWB11614* which were located between markers *KASP-IWB47525*and *KASP-IWB13648* were included (Table S2).

**Fig. 3:**
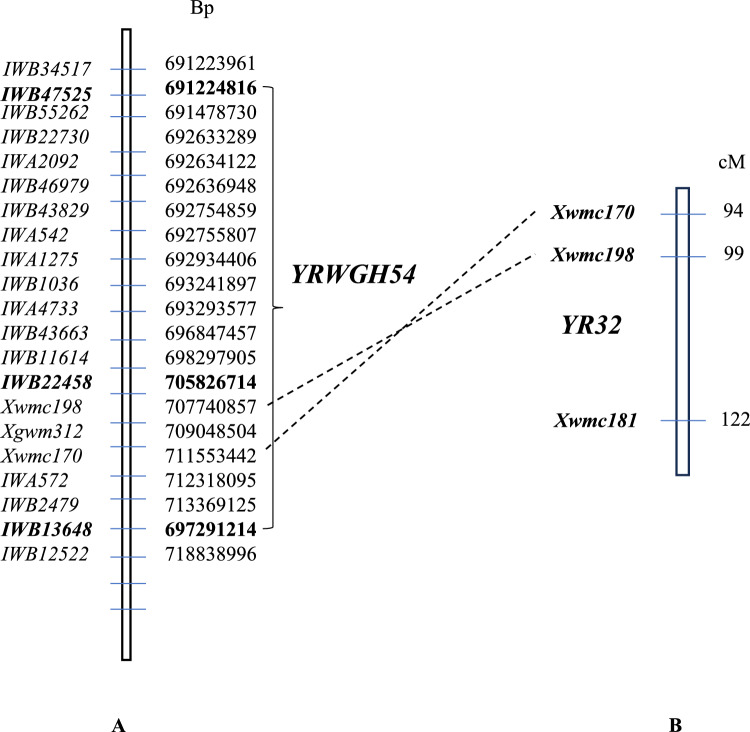
(**A**) Fine mapping of WGH54/AvS (24 Recombinants F_3_) showing marker position based on fine mapping (on left side) and marker position in CS v1.0 reference sequence (right side) (**B**). Map showing position of *YR32* as shown in Eriksen et al.^22^. SSR markers linked to *YR32* and their position on CS reference sequence V1.0 is shown by dotted line.

Eriksen et al.,^22^ previously mapped stripe rust resistance gene *YR32* on chromosome 2AL and reported linkage of three SSR markers *Xwmc198*, *Xgwm312* and *Xwmc170* with this gene. These SSR markers map at positions 707Mbp, 709Mbp and 711Mbp, respectively, on the CS Ref V1.0, which places them distal to marker *KASP-IWB13648* at 697Mbp. These SSR markers were also included in genotyping. Figure 3A, B and Table S2 show the genotypes and phenotypes of selected recombinants. SSR markers *Xwmc198*, *Xgwm312* and *Xwmc170* were mapped proximal to *KASP-IWB13648* suggesting rearrangements in the region when compared to CS reference sequence. The marker *KASP-IWB22458* which is placed at 705Mbp on the CS reference V1.0 flanked the region carrying *YrWGH54*. While the *YR32* linked SSRs were placed distal to the recombining marker *KASP-IWB22458*, with rearrangements in *YRWGH54* region in comparison to the CS reference V1.0, it is possible that *YRWGH54* and *YR32* are the same gene.

The initial pathotyping with *Pst* 134 E16A + 17 + Yr27 + and its variants could not distinguish between the *YRWGH54* and Carstens V (*YR32*). Both *YR32* and *YRWGH54* lines were phenotyped against pathotypes 239 E237A-Yr17 + Yr33 + and 198 E16A + YrJ + YrT + Yr17 + . Table 1 shows the infection types of *YRWGH54* and *YR32* against different *Pst* pathotypes. While *Pst* pathotypes 134 E16A + Yr17 + Yr27 + and 198 E16 A + YrJ + YrT + Yr17 + were avirulent on both *YR32* and *YRWGH54*, the *Pst* pathotype 239 E237A-Yr17 + Yr33 + was virulent on both WGH54 (*YRWGH54*) and Carstens V (*YR32*). This similar pathotypic specificity suggested that *YrWGH54* and *Yr32* may represent the same locus.

Polymorphism of *YRWGH54* linked molecular markers.

Thirty KASP markers linked to *YRWGH54* (Table S3) were used on a set of wheat varieties to look for their suitability for marker assisted breeding. Markers *KASP-IWB22458* and *KASP-IWB13648*, which recombine on the distal end of the locus were found to be most suitable and were further validated on 73 randomly selected wheat varieties (Table S3, Fig. 2). Two of these 70 varieties showed the presence of both these markers and suggest that they may carry *YRWGH54* and or false positives. While previously identified SSR markers linked to *YR32* also map in the region, the high throughput nature of KASP markers make them more suitable for MAS.

## Discussion

The evolution of the new *Pst* pathotypes and their rapid adaptation leads to a shortened field life of varieties carrying ASR genes^23^. For example, in 2003 the rapid spread and evolution of *Pst* pathotype 134 E16A + in eastern Australia resulted in new derivative pathotypes carrying virulence for *YR10*, *YR17*, *YRJ*, *YRT*, *YR24* and *YR27* within a decade of its appearance^1^. The occurrence of such events necessitates the identification of new genes for stripe rust resistance. Pyramiding of widely effective two or more ASR genes into new cultivars can achieve long-lasting control of rust diseases^5,24,25^. Additionally combining ASR and APR genes is a better strategy^5,25^. Pyramiding of stripe rust resistance genes has been previously reported in wheat. Pakeerathan et al.,^26^ developed Aus27969/Avocet S RILs that carried *YR82* and *YR29* in combination and produced lower stripe rust responses than those exhibited by RILs carrying these two genes singly. Chhetri et al.,^27^ reported combination of ASR gene *YR58* with the APR gene *YR46* for enhanced resistance. Similarly, Qie et al.,^6^ reported pyramiding of *YR64* and *YR15*, both on chromosome 1BS, for developing cultivars with potentially durable and high level of resistance.

In the current study, we characterized an all-stage stripe rust resistance locus, *YrWGH54*, in a Glossy Huguenot (Durum wheat)/Westonia-derived hexaploid wheat line WGH54. High-density genome-wide 90 k SNP array^20^ was used for genotyping and *YrWGH54* was initially mapped on the long arm of chromosome 2A in a 58Mbp region. Subsequent analysis identified recombinants, and resistance locus was mapped to a 3.0Mbp region on CS v1.0 sequence flanked by markers *KASP*-*IWB47525* and *KASP*-*IWB13648*. These markers were physically located in Chinese spring V1.0 sequence at positions 691Mbp and 697Mbp, respectively. However, subsequent analysis with additional markers showed rearrangements in WGH54 with respect to CS sequence V1.0 with placement of marker *KASP-IWB13648* distal to marker *KASP-IWB22458* which maps at 705Mbp in CS. Genomic rearrangements at rust resistance loci with respect to CS reference sequence and other wheat pan genome databases have been documented earlier. Sharma et al.^28^ showed rearrangements including deletion at *YrV1* locus when compared to CS sequence.

The long arm of chromosome 2A has previously been known to carry ASR genes for stripe rust. Genes *YR1*^29^, *YRxy2*^30^, *YRJ22*^31^ and *YR32*^22^ have been mapped on long arm of chromosome 2A earlier. Bariana and McIntosh^32^ mapped *YR1* to the distal end of long arm of chromosome 2A. *YRJ22*-linked to markers *Xwmc658* and *IWA1348* were located at 763Mbp and 771Mbp positions, respectively. *YR32*-linked to SSR markers *Xwmc198*, *Xgwm312* and *Xwmc170* were positioned at 707Mbp, 709Mbp and 711Mbp positions, respectively. In the present study, we mapped *YRWGH54* on chromosome 2A in the region where *YR32* has been mapped^22^. Comparison of maps for *YRWGH54* and *YR32* showed chromosomal rearrangement (Fig. 3 A, B) as shown by inversion of SSR marker *Xwmc170* and *Xwmc198* positions with respect to CS v1.0 reference sequence. However, the location of these genes and their virulence profile strongly suggests them being the same gene.

While *YRWGH54* may be the same as *YR32*, it provides high level of resistance to both pre and post 2002 *Pst* pathotypes in Australia, except *Pst* 239 E237A-17 + 33 + , and can be an excellent gene for stacking with other genes to develop wheat cultivars with potentially durable resistance.

This study related resistance in a durum wheat Glossy Huguenot with the *YR32* carrying winter bread wheat cultivar Carstens V. The association of markers *KASP-IWB22458* and *KASP-IWB13648* with *YRWGH54* and/or *YR32* will be useful for marker-assisted pyramiding of this gene with other marker-tagged ASR and APR loci.

## Supplementary Information

Supplementary Information.

## Data Availability

All data and materials are available upon request from corresponding author.
